# From pre-registration to publication: a non-technical primer for conducting a meta-analysis to synthesize correlational data

**DOI:** 10.3389/fpsyg.2015.01549

**Published:** 2015-10-08

**Authors:** Daniel S. Quintana

**Affiliations:** NORMENT, KG Jebsen Centre for Psychosis Research, Division of Mental Health and Addiction, Oslo University Hospital, University of OsloOslo, Norway

**Keywords:** meta-analysis, primer, methods, pre-registration, statistics, publication bias

## Abstract

Meta-analysis synthesizes a body of research investigating a common research question. Outcomes from meta-analyses provide a more objective and transparent summary of a research area than traditional narrative reviews. Moreover, they are often used to support research grant applications, guide clinical practice, and direct health policy. The aim of this article is to provide a practical and non-technical guide for psychological scientists that outlines the steps involved in planning and performing a meta-analysis of correlational datasets. I provide a supplementary R script to demonstrate each analytical step described in the paper, which is readily adaptable for researchers to use for their analyses. While the worked example is the analysis of a correlational dataset, the general meta-analytic process described in this paper is applicable for all types of effect sizes. I also emphasize the importance of meta-analysis protocols and pre-registration to improve transparency and help avoid unintended duplication. An improved understanding this tool will not only help scientists to conduct their own meta-analyses but also improve their evaluation of published meta-analyses.

A meta-analysis is a statistical integration of evidence from multiple studies that address a common research question. By extracting effect sizes and measures of variance, numerous outcomes can be combined to compute a summary effect size. Meta-analyses are commonly used to support research grant applications, treatment guidelines, and health policy. Moreover, meta-analytic outcomes are often used to summarize a research area in an effort to better direct future work. There is growing interest in using meta-analysis within the psychological sciences (**Figure 1**). This trend is likely to continue given exponential increases in published research (Bornmann and Mutz, 2015) and the wider availability of software and scripts for computing meta-analyses. Prior treatments have outlined the protocol (Shamseer et al., 2015) and pre-registration (Stewart et al., 2012) process, the theory behind conducting meta-analysis with vignettes (Viechtbauer, 2010), and guidelines for reporting meta-analysis (Moher et al., 2009). However, it appears these approaches have yet to be combined in a single resource targeting psychological scientists.

**FIGURE 1:**
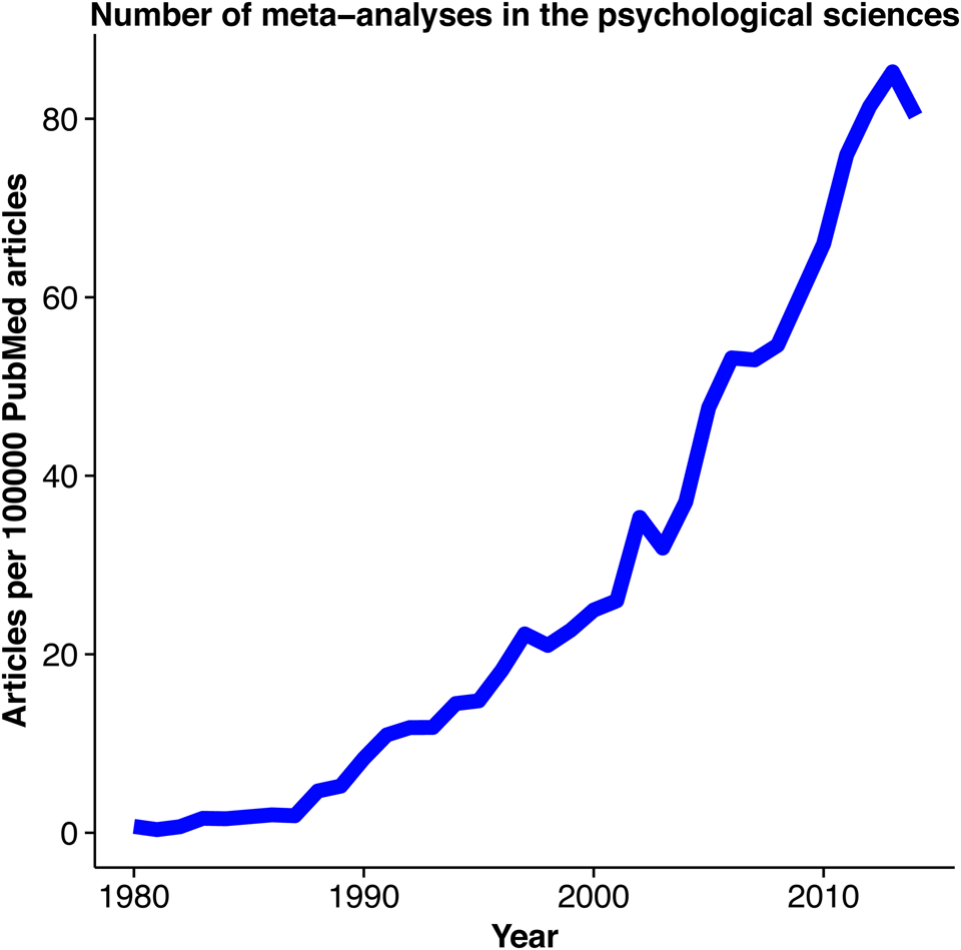
**Meta-analysis in the psychological sciences**. An illustration of the increasing interest in performing meta-analyses in the psychological sciences. PubMed data was collected on the number of articles containing the search terms “psychology” and “meta-analysis” published between 1980 and 2014 per 100,000 PubMed articles. Data was collected using the ‘RISmed’ R package.

The goal of this article is to provide a brief non-technical primer to guide the reader through this process, from pre-registration to the publication of results. Over half of the 25 journals that publish the most meta-analyses in psychology (**Figure 2**) recommend the use of the Preferred Reporting Items for Systematic Reviews and Meta-Analyses guidelines (PRISMA; Moher et al., 2009), or the related Meta-Analysis Reporting Standards (MARS; APA, 2008). Therefore, this review will demonstrate how to conduct a meta-analysis following PRISMA guidelines. Example analyses will be demonstrated using packages within the freely available *R* statistical environment (R Development Core Team, 2015). Performing a meta-analysis with *R* can appear daunting to those accustomed to “point and click” statistical environments such as SPSS and SAS. Thus, a supplementary step-by-step script illustrating the analytic procedures described in this paper, which requires only a rudimentary understanding of R, has been provided. Ultimately, the intention of this article is to improve understanding of meta-analytical procedures, and also better prepare the reader to evaluate published meta-analyses. For a more in-depth and technical treatment of step-by-step meta-analysis methods, a range of excellent resources are available (Lipsey and Wilson, 2001; Hunter and Schmidt, 2004; Cooper, 2009).

**FIGURE 2:**
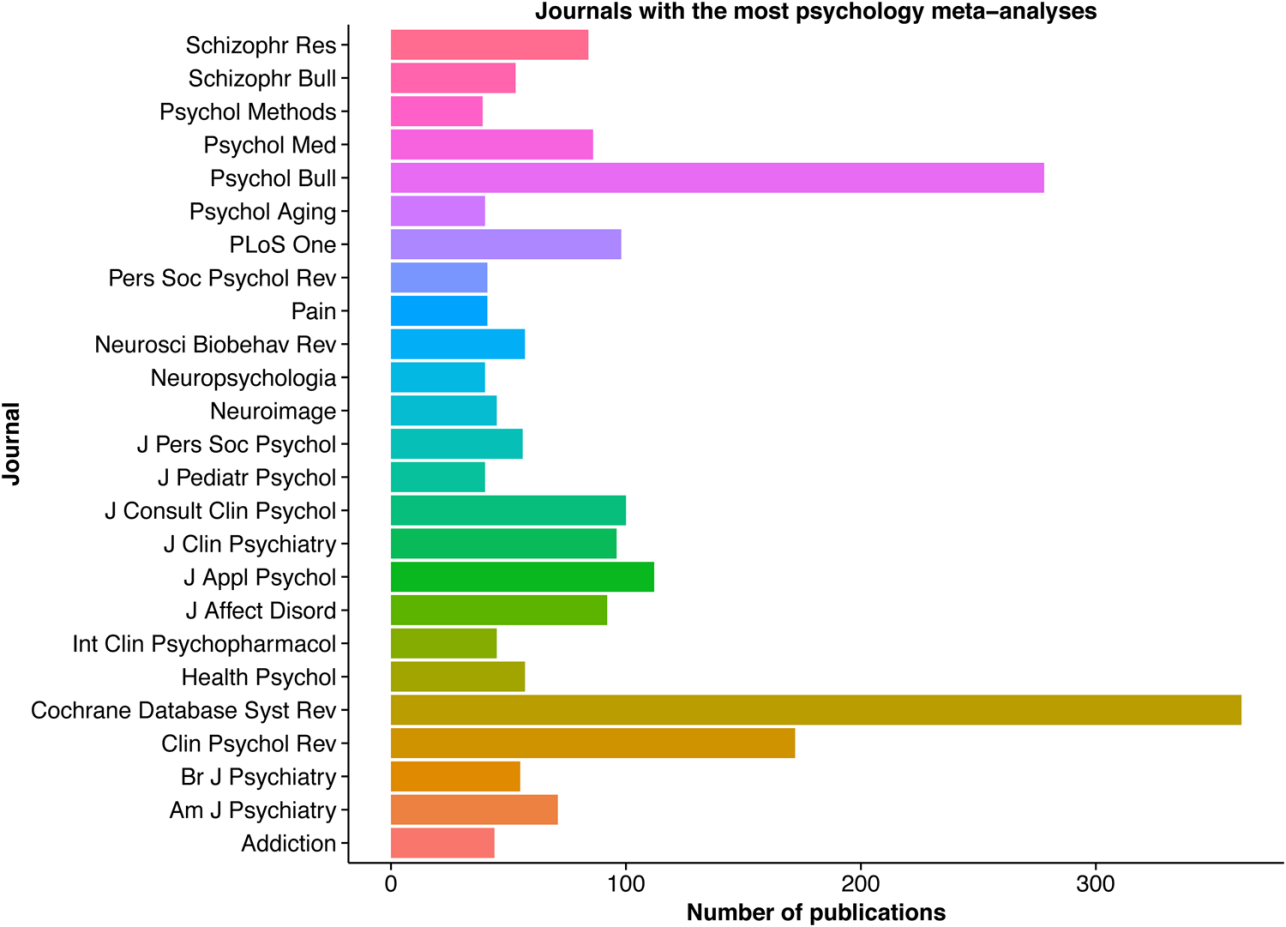
**Psychology journals that publish the greatest number of meta-analyses**. The number of publications containing the keywords “psychology” and “meta-analysis” for the 25 journals with the most meta-analysis in psychology. Data was collected using the ‘RISmed’ R package.

## Meta-analysis Protocol and Pre-registration

The chief benefits of pre-registering a meta-analysis protocol are twofold. Firstly, the pre-registration process compels the researcher to formulate a study rationale for a specific research question, which is the foundation of a good systematic review (Counsell, 1997). Secondly, pre-registration helps avoids bias by providing evidence of *a priori* analysis intentions. Like other types of empirical research, meta-analysis is susceptible to hypothesizing after the results are known, otherwise known as “HARKing” (Kerr, 1998). In the case of meta-analysis, inclusion criteria can be adjusted after results are known to accommodate sought-after results or reduce evidence of publication bias. Alternatively, the analysis could be left unpublished due to non-significant results (Tricco et al., 2009). These issues are especially relevant if researchers have a material or intellectual conflict of interest. Pre-registration of clinical trials is a requirement for submission to almost all peer-reviewed journals; however, few journals explicitly require meta-analysis registration. Indeed, the pre-registration of meta-analysis is arguably more important than clinical trial pre-registration as meta-analyses are often used to guide treatment practice and health policy.

The PRISMA protocol (PRISMA-P) guidelines (Shamseer et al., 2015) provide a framework for reporting meta-analysis protocols. These guidelines recommend that protocols include details such as study rationale, study eligibility criteria, search strategy, moderator variables, risk of bias, and statistical approach. As meta-analyses are iterative processes, protocols are likely to change over time. Indeed, over 20% of meta-analyses make changes to original protocols (Dwan et al., 2011). By having a record of a protocol prior to analysis, these changes are transparent. Any deviations from the original protocol can be stated in the methods section of the paper. Study protocols can be submitted as preprints (e.g., PeerJ PrePrints, Open Science Framework, bioRxiv) or submitted as a peer-reviewed article to open access journals that accept study protocols (e.g., *BMC Psychology, Systematic Reviews*). Meta-analyses can also be registered in the PROSPERO database^1^, which guidelines formed the basis for the PRISMA-P checklist. In addition to the PRISMA-P guidelines, online resources are available that provide information on meta-analysis pre-registration specific to social science^2^ and medicine^3^. Although most journals do not explicitly state that meta-analysis registration is a requirement, many require the submission of a PRISMA checklist, which includes a protocol and study registration. Additionally, pre-registration may help avoid unintended meta-analysis duplication. Checking whether other researchers are in the process of conducting a similar meta-analysis can potentially save valuable resources.

## Literature Search and Data Collection

One of the most important steps of a meta-analysis is data collection. For an efficient database search, appropriate keywords and search limits need to be identified. Key articles will be overlooked if these search terms are too narrow. On the other hand, overly broad search terms will return a large volume of literature that may be irrelevant for the analysis. Nonetheless, it is better to slightly overshoot the mark to avoid missing important articles. The use of Boolean operators and search limits can assist the literature search (for examples, see Sood et al., 2004; Vincent et al., 2006). A number of databases are available (e.g., PubMed, Embase, PsychInfo), however, it is up to the researcher to choose the most appropriate sources for their research area. Indeed, many scientists use duplicate search terms within two or more databases to cover multiple sources. The reference lists of eligible studies can also be searched for eligible studies (i.e., snowballing). The initial search may return a large volume of studies. Quite often, the abstract or the title of the manuscript reveals that the study is not eligible for inclusion, based on the pre-specified criteria. These studies can be discarded. However, if it appears that the study may be eligible (or even if there is some doubt) the full paper can be retained for closer inspection. The references lists of eligible articles can also be searched for any relevant articles. These search results need to be detailed in a PRIMSA flow diagram (Moher et al., 2009), which details the flow of information through all stages of the review. Thus, it is important to note how many studies were returned after using the specified search terms and how many of these studies were discarded, and for what reason. The search terms and strategy should be specific enough for a reader to reproduce the search. The date range of studies, along with the date (or date period) the search was conducted should also be provided.

A data collection form provides a standardized means of collecting data from eligible studies. For a meta-analysis of correlational data, effect size information is usually collected as Pearson’s *r* statistic. Partial correlations are often reported in research, however, these may inflate relationships in comparison to zero-order correlations (Cramer, 2003). Moreover, the partialed out variables will likely vary from study-to-study. As a consequence, many meta-analyses exclude partial correlations from their analysis (e.g., Klassen and Tze, 2014; Jim et al., 2015; Slaughter et al., 2015). Thus, study authors should be contacted to provide missing data or zero-order correlations. As a final resort, plot digitizers (e.g., Gross et al., 2014) can be used to scrape data points from scatterplots (if available) for the calculation of Pearson’s *r*. Data reporting important study characteristics that may moderate effects, such as the mean age of participants, should also be collected. Piloting the information required by randomly selecting a few eligible studies during the early stages of meta-analysis planning will help refine the form. A measure of study quality can also be included in these forms to assess the quality of evidence from each study. There are more than 80 tools available to assess the quality and risk of bias in observational studies (Sanderson et al., 2007) reflecting the diversity of research approaches between fields. These tools usually include an assessment of how dependent variables were measured, appropriate selection of participants, and appropriate control for confounding factors. Other quality measures that may be more relevant for correlational studies include sample size, psychometric properties, and reporting of methods. In many cases, such as the example meta-analysis used in this paper (Molloy et al., 2014), a bespoke quality tool is developed integrating various criteria suited to the meta-analysis research question. Along with providing a measure of overall risk of bias – which is one of the checklist items in PRISMA – this can also be assessed as a moderator for the meta-analysis.

A final consideration is whether to include studies from the gray literature, which is defined as research that has not been formally published. This type of literature includes conference abstracts, dissertations, and pre-prints. While the inclusion of gray literature reduces the risk of publication bias, the methodological quality of the work is often (but not always) lower than formally published work (Egger et al., 2003). Reports from conference proceedings, which are the most common source of gray literature (McAuley et al., 2000), are poorly reported (Hopewell and Clarke, 2005) and data in the subsequent publication is often inconsistent, with differences observed in almost 20% of published studies (Bhandari et al., 2002). The meta-analyst needs to consider the main sources of where they expect studies to reside. While the gray literature has been traditionally difficult to access in some cases, it is becoming increasingly accessible with many universities now posting dissertations in online repositories. Thus, the advantages and disadvantages of including gray literature need to be considered. Differences between fields and research questions preclude any blanket recommendations. Regardless, meta-analyses should explicitly detail search strategy in the study protocol and methods.

## Analysis

Various tools are available for performing a meta-analysis, such as Comprehensive Meta-Analysis (Borenstein et al., 2005) and SPSS syntax files (Field and Gillett, 2010). For this review, I will use the “metafor” (Viechtbauer, 2010) and “robumeta” (Fisher and Tipton, 2015) packages for *R* (R Development Core Team, 2015). *R* is an ideal software package to perform meta-analyses because it is freely available and the scripts used can be easily shared and reproduced. For illustration, data from a meta-analysis of sixteen studies (Molloy et al., 2014) that investigate the association between conscientiousness and medication adherence will be analyzed (**Table 1**). The dataset includes correlations, study sample sizes, and a range of continuous (e.g., mean age) and categorical variables (e.g., type of conscientiousness measure used) that can assessed as potential moderators. The data from this meta-analysis, along with analysis examples, are included in the metafor package (Viechtbauer, 2010). The script associated with this paper (also available at: http://github.com/dsquintana/corr_meta) details all aspects of the analysis described herein, which readers can adapt to perform their own meta-analyses of correlational data.

**Table 1:** Example meta-analysis data (Molloy et al., 2014).

Study id	Authors	Publication year	Study sample size	Correlation	Variables controlled	Study design	Adherence measure	Conscientiousness measure	Mean age	Methodological quality
1	Axelsson et al.	2009	109	0.187	None	Cross-sectional	Self-report	other	22	1
2	Axelsson et al.	2011	749	0.162	None	Cross-sectional	Self-report	NEO	53.59	1
3	Bruce et al.	2010	55	0.34	None	Prospective	Other	NEO	43.36	2
4	Christensen et al.	1999	107	0.32	None	Cross-sectional	Self-report	other	41.7	1
5	Christensen and Smith	1995	72	0.27	None	Prospective	Other	NEO	46.39	2
6	Cohen et al.	2004	65	0	None	Prospective	Other	NEO	41.2	2
7	Dobbels et al.	2005	174	0.175	None	Cross-sectional	Self-report	NEO	52.3	1
8	Ediger et al.	2007	326	0.05	Multiple	Prospective	Self-report	NEO	41	3
9	Insel et al.	2006	58	0.26	None	Prospective	Other	other	77	2
10	Jerant et al.	2011	771	0.01	Multiple	Prospective	Other	NEO	78.6	3
11	Moran et al.	1997	56	-0.09	Multiple	Prospective	Other	NEO	57.2	2
12	O’Cleirigh et al.	2007	91	0.37	None	Prospective	Self-report	NEO	37.9	2
13	Penedo et al.	2003	116	0	None	Cross-sectional	Self-report	NEO	39.2	1
14	Quine et al.	2012	537	0.15	None	Prospective	Self-report	other	69	2
15	Stilley et al.	2004	158	0.24	None	Prospective	Other	NEO	46.2	3
16	Wiebe and Christensen	1997	65	0.04	None	Prospective	Other	NEO	56	1

The first analysis step is entering data from collection forms into a .csv file for analysis in R. As Pearson’s *r* is not normally distributed, these values are converted into Fisher’s *z* scale. However, before the meta-analysis can be performed, the meta-analysis model needs to be specified. Two models are commonly adopted in meta-analysis: the fixed- and random-effects models. The selection of these models center around assumptions of study homogeneity, that is, how much of the variation of studies can be attributed to variation in the true effect sizes. Variation is derived from both random error and true study heterogeneity. The fixed-effects model assumes that all studies are from a single common population, tested under similar conditions. For instance, a series of studies done in the same laboratory, drawing from the same population may qualify for the fixed-effects model. As the fixed-effects model does not account for study heterogeneity, it can overestimate the summary effect sizes if studies are not drawn from the same population. Thus, if studies are drawn from different populations, the random-effects model should be used. The random-effects model also provides less study weight to larger studies with less variance. As a result, the calculated confidence interval (CI) is much wider than the CI that would be generated by using a fixed-effects model. Even if the random-effects model is applied to homogenous studies, it will calculate a CI equivalent to the fixed-effects model. After performing the meta-analytic calculations, Fisher’s *z* should be converted back to Pearson’s *r* to for reporting the average correlation and 95% CI. Performing the analysis of the example data reveals a summary correlation and 95% CI indicative of a significant, but modest, relationship between conscientiousness and medication adherence [*r* = 0.15; 95% CI (0.09, 0.21), *p* < 0.0001].

## Study Heterogeneity

There are two sources of variation in observed effects: within-study error and real heterogeneity in effect size. For the purposes of meta-analysis we are interested in the true heterogeneity in effect sizes. Calculating the *Q*-statistic, which is the ratio of observed variation to within-study variance, can reveal how much of the overall heterogeneity can be attributed to true between-studies variation. The *Q*-statistic is a null hypothesis significance test (NHST) that evaluates the null hypothesis that all studies are examining the same effect. Consequently, a statistically significant *Q*-statistic indicates that the included studies do not share a common effect size. Like any other NHST, however, a non-significant *Q*-statistic does not provide evidence that studies are homogeneous. Further, the *Q*-statistic is prone to underestimating heterogeneity in small samples and overestimating for large samples (Higgins et al., 2003). The related *I*^2^ statistic is a percentage that represents the proportion of observed variation that can be attributed to the actual difference between studies, rather than within-study variance. *I*^2^ thresholds have been proposed (Higgins et al., 2003), with 25, 50, and 75% representing low, moderate, and high variance, respectively. The two main advantages of *I*^2^, compared to the *Q*-statistic, are that it is not sensitive to the number of studies included and that CIs can also be calculated. Meta-analyses comprising heterogeneous studies provide less weight to larger studies with smaller variance. Tau-squared can also be used to estimate the total amount of study heterogeneity in random-effects models. When Tau-squared is zero this is indicative of no heterogeneity. In the example data, *I*^2^ was 61.73% (95% CI; 25.28, 88.25) – which represents moderate-to-high variance – the *Q*-statistic was 38.16 (*p* = 0.001), and tau-squared was 0.008 (95% CI; 0.002, 0.038).

**FIGURE 3:**
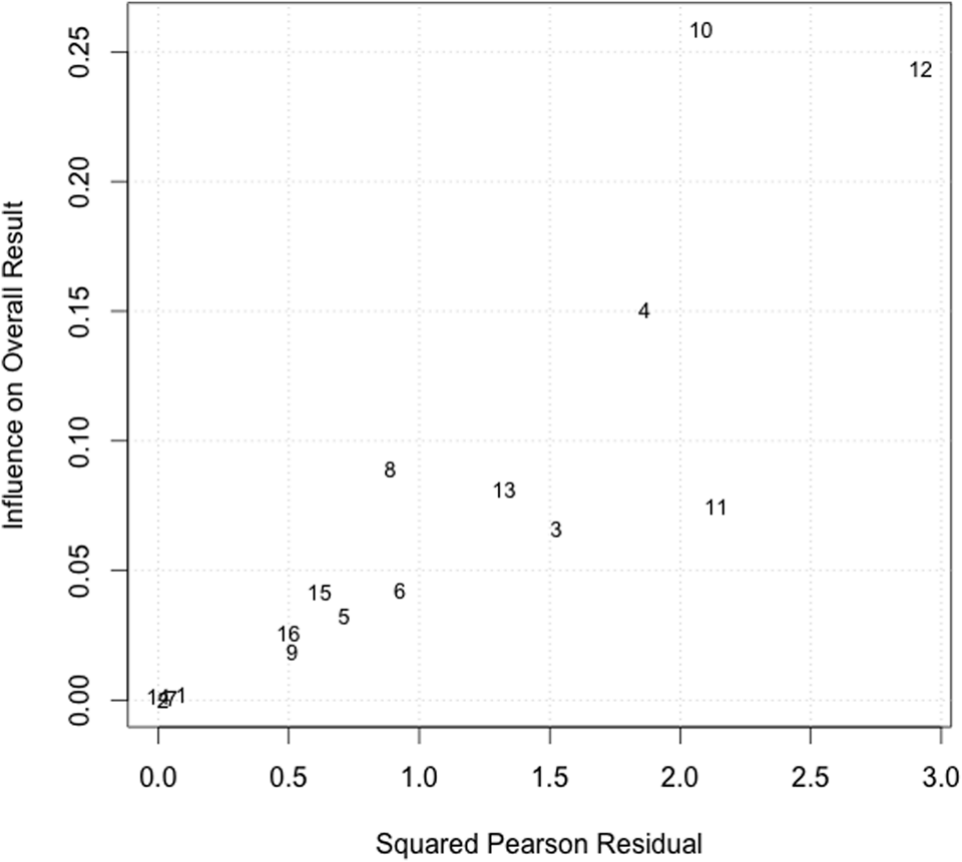
**Baujat plot to identify studies contributing to heterogeneity**. Each study is represented by a study id number. Studies located in the top right quadrant have both a greater influence on the overall result and contribute most to study heterogeneity.

Although these tests provide evidence for heterogeneity, they do not provide any indication of which studies that may be disproportionately influencing heterogeneity. Baujat et al. (2002) have proposed a plot to identify studies that excessively contribute to heterogeneity and the overall result. The plot’s horizontal axis illustrates study heterogeneity whereas the vertical axis illustrates the influence of a study on the overall result. Studies that fall to the top right quadrant of the plot contribute most to both these factors. Examining the Bajaut plot generated from the example dataset reveals three studies that contribute to both of these factors (**Figure 3**). A closer look at the characteristics of these studies may reveal moderating variables that contribute to heterogeneity. A set of diagnostics derived from standard linear regression are also available within the metafor package to identify potential outliers and influential cases, which can also influence observed heterogeneity (Viechtbauer and Cheung, 2010). None of the studies in the example dataset were identified as potential outliers.

## Forest Plots

**FIGURE 4:**
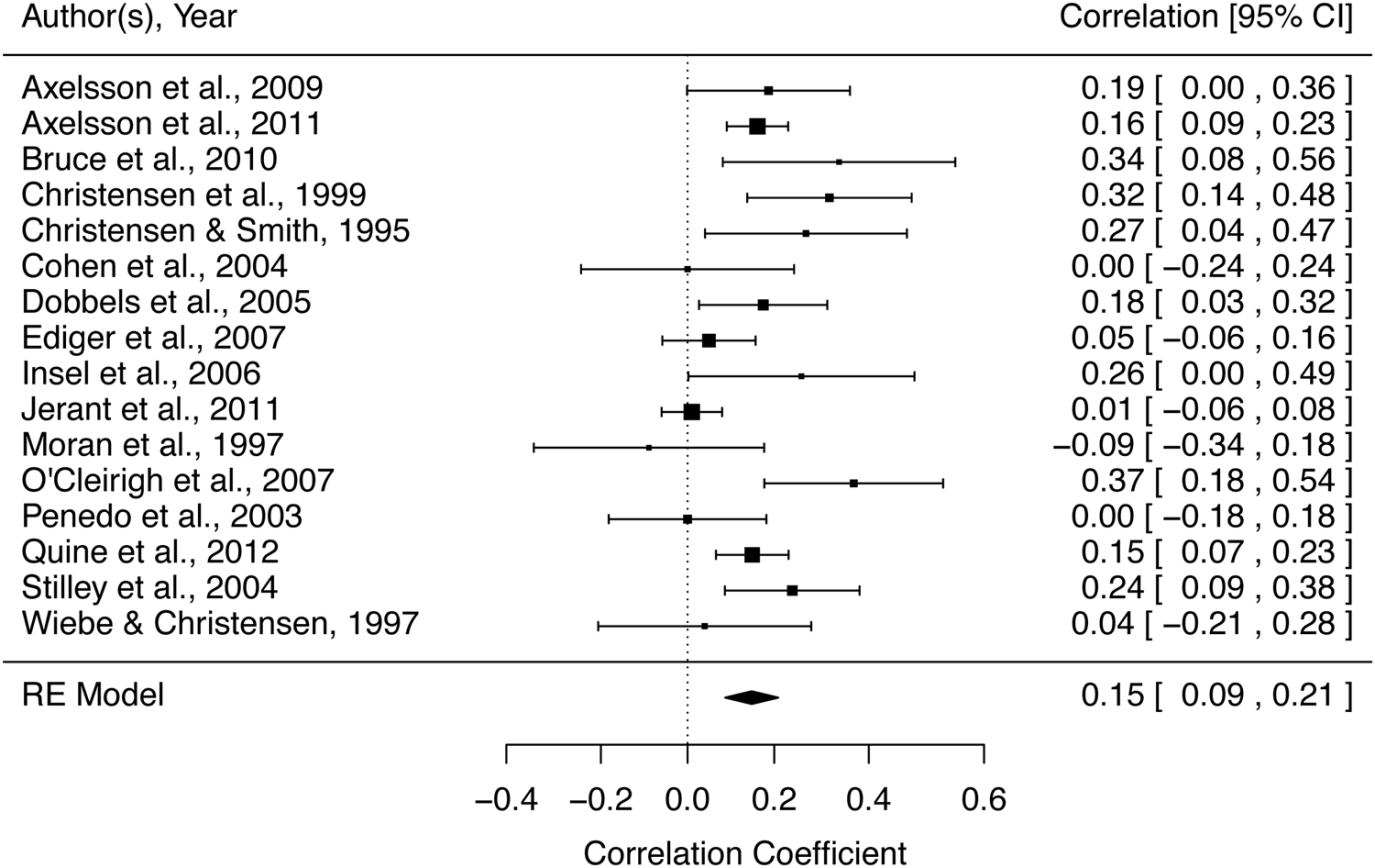
**Forest plot of example data**. Summary of example data investigating the relationship between conscientiousness and medication adherence. Each study included in the meta-analysis is represented by a point estimate, which is bounded by a 95% CI. The summary effect size is displayed as a polygon at the bottom of the plot, with the width of the polygon representing the 95% CI.

Forest plots visualize the effect sizes and CIs from the included studies, along with the computed summary effect size. **Figure 4** illustrates the forest plot calculated from the example data. Each study is represented by a point estimate, which is bounded by a CI for the effect. The summary effect size is represented by the polygon at the bottom of the plot, with the width of the polygon representing the 95% CI. Consistent with the high *I*^2^ and significant *Q*-statistic, the forest plot illustrates a sample of heterogeneous studies. Studies with larger squares have contributed more to the summary effect size compared to other studies. In a random-effects model, the size of the square is related to both the CI and between-studies variance.

## Publication Bias

**FIGURE 5:**
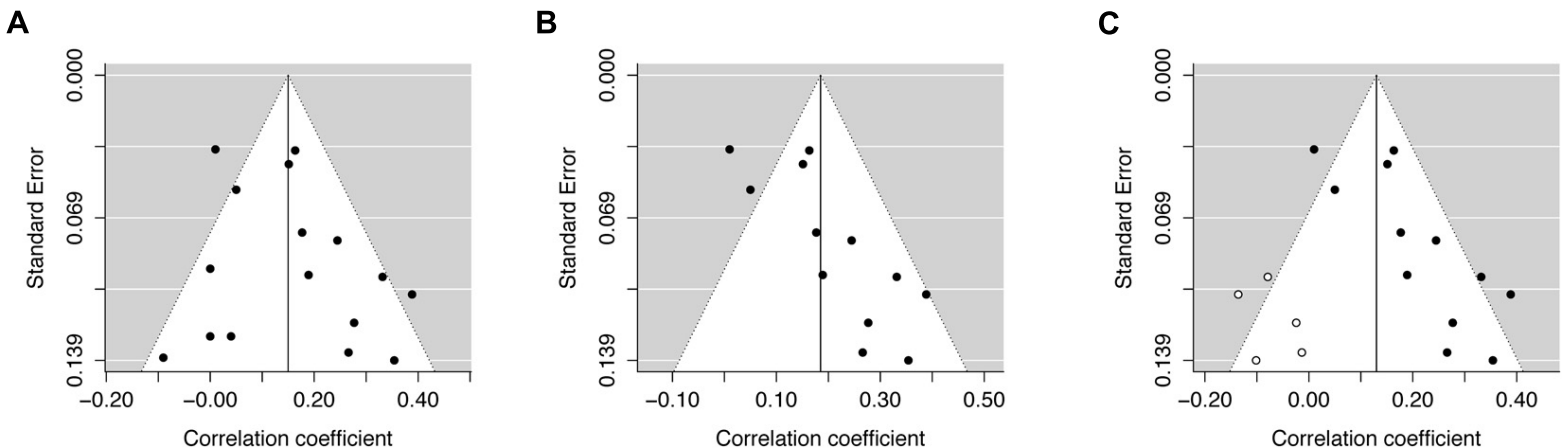
**Funnel plots to illustrate publication bias**. Funnel plot **(A)** includes all 16 studies from Molloy et al. (2014). This plot illustrates symmetry (i.e., points fall on both sides of the summary effect size). Egger’s regression test (*p* = 0.31) was consistent with this data, as the *p*-value was above 0.05. Funnel plot **(B)** simulates the removal of three studies with small effect sizes and large standard error from the Molloy et al. (2014) dataset. The plot is no longer symmetrical, demonstrating evidence of publication bias. Egger’s regression test (*p* = 0.01) was also consistent with this data, as the *p*-value was below 0.05. The trim and fill procedure imputes missing studies (hollow circles) to create a more symmetrical funnel plot **(C)**.

Publication bias is the phenomenon whereby studies with stronger effects sizes are more likely to be published and subsequently included in a meta-analysis. A funnel plot is a visual tool used to examine potential publication bias in meta-analyses. These plots illustrate the individual effect sizes on the horizontal axis and corresponding standard errors on the vertical axis. Studies with smaller standard errors (usually larger studies) lie closer to the top of the plot. The funnel lines are centerd on the summary effect size, represented by the vertical line, and indicate the degree of spread that would be expected for a given level of standard error. In other words, the effect size of a study with low standard error would not be expected to stray very far from the vertical line. As the vertical line represents the summary of all the studies, these points should be equivalently spread on both sides of the line (**Figure 5A**). Publication bias dictates that studies with non-significant results are less likely to be published. Consequently, if a significant positive effect were found for the summary effect size, for instance, the vertical line would be situated to the right of zero. Any study with a non-significant effect would lie around zero, thus if the funnel is uneven with more positively associated studies to the right of the line this provides evidence for publication bias. **Figure 5B** illustrates this using a simulation of removing three studies from the example dataset. It is important to note that studies often report a significant negative association. If this were the case then missing studies would be situated to the right of the vertical line. Although funnel plots provide a useful visualization for potential publication bias, it is important to consider that asymmetry may reflect other types of bias, such as study quality, location bias, and study size (Egger et al., 1997).

Another weakness of funnel plots is that they only offer a subjective measure of potential publication bias. Two tests are often employed to calculate an objective measure of potential bias. The rank correlation test (Begg and Mazumdar, 1994) evaluates if effect estimates and sampling variances for each study are related. A significant test (*p* < 0.05) is consistent with a non-symmetrical funnel plot. However, the rank correlation test may only have moderate power for smaller meta-analysis (Begg and Mazumdar, 1994; Sterne et al., 2000). An alternative test that is better suited to smaller meta-analyses (<25 studies) is Egger’s regression test (Egger et al., 1997).

In the example data, neither the rank correlation (*p* = 0.39) nor Egger’s regression test (*p* = 0.31) were statistically significant, which is consistent with funnel plot symmetry. However, in the simulated “biased” dataset (see Supplementary Material), the rank correction (*p* = 0.01) and regression (*p* = 0.001) tests were statistically significant, consistent with funnel plot asymmetry and potential publication bias. If there is evidence of publication bias, the trim and fill method can be used (Duval and Tweedie, 2000). This method, which assumes that funnel plot asymmetry is due to publication bias, adjusts a meta-analysis by imputing “missing” studies to increase funnel plot symmetry (**Figure 5C**). This updated meta-analysis with imputed studies should not be used to form conclusions – as these are not real studies – only as an effort to balance out asymmetrical funnel plots. A comparison of **Figures 5A,C** illustrates this as the method is designed to only approximate the missing studies by creating a mirror image of the existing studies. Consistent with prior work (Pham et al., 2001), the trim and fill method using the current data provides a reasonable estimate of how many studies are missing – assuming that the studies in the example meta-analysis represent all existing studies. As shown previously (Terrin et al., 2003), the trim and fill method may slightly overestimate missing studies (four studies were removed, but five were imputed). Nevertheless, the method can be used a form of sensitivity analysis to assess the potential impact of these probable studies on the summary effect size.

## Moderator Analysis

Moderating variables contribute to some of the observed variance. Thus, a moderator analysis can be conducted to determine the source of heterogeneity and how much this contributes to the observed variability of effect sizes between studies. Moderating variables can either be continuous or categorical variables. For example, a moderator analysis using a meta-regression model can be conducted to examine the influence of mean age on the Molloy et al. (2014) dataset. Computing this analysis reveals that age did not have a moderating effect [*Q*(1) = 1.43, *p* = 0.23]. Additionally, the moderating effect of methodological quality can be examined. Analysis of the example data indicated that methodological quality also did not moderate the correlation [*Q*(1) = 0.64, *p* = 0.42]. However, moderator analysis suggests that the variable categorizing whether studies controlling for variables (yes/no) was a significant moderator [*Q*(1) = 20.12, *p* < 0.0001]. While there may be other unidentified sources of study heterogeneity, the data indicate that controlling for variables within studies contributes to the overall observed heterogeneity.

## Accounting for Multiple Effect Sizes from Individual Studies

If more than one set of data has been collected from the same study, the within-subject statistical dependency of these effect sizes should be accounted for due to issues of statistical dependency (Hunter and Schmidt, 2004). There are a number of approaches to this issue. The most straightforward procedure is to only collect one effect size per study using pre-specified criteria (e.g., Chalmers et al., 2014; Alvares et al., in press). Alternatively, effect sizes can be aggregated (See the ‘Agg’ function in the ‘MAc’ *R* package; Del Re and Hoyt, 2010). However, without reported within-study correlations, the researcher has to estimate a level of expected correlations. Robust variance estimation (RVE) can account for non-independent sizes without knowledge of within-study correlations (Hedges et al., 2010). RVE estimators can also be adjusted to better suit smaller meta-analyses (*n* < 40; Tipton, 2015). To illustrate the use of RVE to handle multiple effect sizes, a new simulated dataset has been created with the first three studies from the sample data set treated as if they were three effect sizes reported from a single study (see Supplementary Material). Analysis using RVE reveals a statistically significant point estimate [0.15; 95% CI (0.08, 0.22), *p* = 0.001]

## Data Interpretation and Reporting

The final step of a meta-analysis is data interpretation and write-up. The PRISMA guidelines provide a checklist that includes all the items that should be included when reporting a meta-analysis (Moher et al., 2009). Following this checklist will help ensure the quality of reporting meta-analysis and facilitate improved evaluation of manuscripts. An important point for moderator analysis is that results are not over-interpreted. For instance, performing a moderator analysis of the effect of gender may reveal a difference, however, there could other unidentified study characteristics that can better explain the moderating effects. In other words, moderator analysis does not specifically target a single variable, but rather, a set of studies that happen to share that variable. Relatedly, the absence of a statistically significant effect does not provide evidence for the null hypothesis (i.e., that there is no relationship between two variables). Thus, caution is required when interpreting non-significant summary effect sizes. Finally, the *R* script used for analysis can also be provided as supplementary material to aid reproducibility.

## General Discussion

The purpose of this article is to provide a non-technical primer for conducting meta-analyses of correlational data, following gold-standard guidelines. Meta-analysis is an effective method to synthesize data, which can meaningfully increase statistical precision even from as little as two or three studies. Although meta-analysis is a valuable tool, it is seldom taught in undergraduate statistics courses (Cumming, 2006). This paper demonstrates each step of the analysis for researchers that are unfamiliar with meta-analytic methods, using freely accessible software. The supplementary script provides the necessary code to carry out the analyses described in the paper. Methods for data visualization, identifying studies that may be excessively influencing sample heterogeneity, and combining multiple effect sizes from individual studies are also discussed. Some caveats for meta-analysis data interpretation in regards to publication bias and moderator analysis are also described. I have limited this article to correlational studies for the sake of brevity and focus. Future research would benefit from similar non-technical primers with supplementary scripts on other types of effect sizes, such as *F* or *t*-tests. However, other than the analysis section, this paper is broadly applicable for the meta-analysis of other effect size types.

Up to 63% of psychological scientists anonymously admit to questionable research practices (John et al., 2012). These practices include removing data points and reanalysing data, failing to report all measures analyzed, and HARKing. Such behavior has likely contributed to the low rates of successful replication observed in psychology (Open Science Collaboration, 2015). The pre-registration of clinical trial protocols has become standard. In contrast, less than 10% of meta-analyses refer to a study protocol, let alone make the protocols publically available (Moher et al., 2007). Thus, meta-analyses pre-registration would markedly improve the transparency of meta-analyses and the confidence of reported findings.

## Conflict of Interest Statement

The author declares that the research was conducted in the absence of any commercial or financial relationships that could be construed as a potential conflict of interest.

## Footnotes

1 http://www.crd.york.ac.uk/PROSPERO/

2 http://www.campbellcollaboration.org/int_dev_our_group/how_to_register.php

3 http://community.cochrane.org/cochrane-reviews/proposing-new-reviews.
