# Supplementary material for: From pre-registration to publication: a non-technical primer for conducting a meta-analysis to synthesize correlational data
Source: Front Psychol. 2015 Oct 8;6:1549. doi: 10.3389/fpsyg.2015.01549 (PMC4597034; doi:10.3389/fpsyg.2015.01549)
Supplement: Supplementary file 1 [file Data_Sheet_1.DOCX]

Supplementary Material

From pre-registration to publication: a nontechnical primer for conducting a meta-analysis to synthesize correlational data

Daniel S. Quintana ^*^

*** Correspondence:** Daniel S. Quintana: daniel.quintana@medisin.uio.no

# Three files are included as supplementary material;

**Data sheet 2:** The supplementary R script that can be copy and pasted into R. This is also available at <http://github.com/dsquintana/corr_meta>

**Data sheet 3:** A simulated “biased” dataset to demonstrate the trim and fill procedure. To follow the R script, this .csv file needs to be saved in the R working directory using the file name “dat_bias.csv”.

**Data sheet 4:** A simulated dataset that aggregates the first 3 studies from the original dataset to demonstrate how to work with multiple effect sizes from a single study. To follow the R script, this .csv file needs to be saved in the R working directory using the file name “dat_mes.csv”.
