# Supplementary material for: From pre-registration to publication: a non-technical primer for conducting a meta-analysis to synthesize correlational data
Source: Front Psychol. 2015 Oct 8;6:1549. doi: 10.3389/fpsyg.2015.01549 (PMC4597034; doi:10.3389/fpsyg.2015.01549)
Supplement: Supplementary file 2 [file Data_Sheet_2.DOC]

#############################################################################################

## A non-technical primer for conducting a meta-analysis to synthesize correlational data ##

#############################################################################################

# Written by Daniel S. Quintana. If you have any questions or comments, contact me over via email daniel.quintana@medisin.uio.no or Twitter @dsquintana.

# Any updates to the script will be posted to http://github.com/dsquintana/corr_meta

###############################

## Install and load packages ##

###############################

install.packages(c("robumeta", "metafor", "dplyr"))

library("robumeta")

library("metafor")

library("dplyr")

######################

## Import your data ##

######################

# For the analyses described in Quintana 2015, the data is included with the metafor package. Use the following the command to load the data. You are creating a new object called "dat" from the Molloy et al., (2014) data.

dat <- get(data(dat.molloy2014))

dat <- mutate(dat, study_id = 1:16) # This adds a study id column

dat <- dat %>% select(study_id, authors:quality) # This brings the study id column to the front

# Take look at the data.

View(dat)

#########################################################################

## Skip next section if you're following the analysis in Quintana 2015 ##

#########################################################################

#You can use a similar procedure to load your own data. However you'll need to use a different command to do this.

#To import your data, follow the instructions below. This will import your data assuming that the file is saved as a csv file in your working directory using the name "yourdata", and that the first row contains variable names. The rest of the guide will use the Molloy et al., 2014 data. If you are using your own data you'll need to replace "dat" file with the name of your dataset.

yourdata <- read.csv("yourdata.csv", header=TRUE)

##################################

## Performing the Meta-analysis ##

##################################

# The first step is to transform r to Z and calculate the corresponding sample variances.

dat <- escalc(measure="ZCOR", ri=ri, ni=ni, data=dat, slab=paste(authors, year, sep=", "))

# This performs the r to z transform from the "dat" dataset and calculates the corresponding sample variances. "ri" represents the correlation coefficients and "ni" represent the sample sizes.

# Lets have a look at the file again, notice the two new variables at the end. The "yi" variable is the z score transformation and the "vi" variable is the corresponding estimated sampling variance.

View(dat)

# Now you're ready to perform the meta-analysis using a random-effects model. The following commands will print out the data and also calculates and print the confidence interval for the amount of heterogeneity (I^2).

res <- rma(yi, vi, data=dat)

res

predict(res, digits=3, transf=transf.ztor)

confint(res)

# The output provides important information to report the meta-analysis, let's look section-by-section at the relevant data.

# "Random-Effects Model (k = 16; tau^2 estimator: REML)"

#This line tells us we've used a random-effects model, with 16 studies (i.e., "k") and that the degree of heterogeneity (tau^2) was calculated using a restricted maximum-likelihood estimator.

# "tau^2 (estimated amount of total heterogeneity): 0.0081 (SE = 0.0055)"

# This line indicates that tau-squared was 0.0081

# "I^2 (total heterogeneity / total variability): 61.73%"

# This line indicates that I^2 was 61.73%. In other words 62.73% of variation reflected actual differences in the population mean. The confidence interval test revealed the 95% confidence interval for this value is 25.27, 88.24.

# "Test for Heterogeneity:

# Q(df = 15) = 38.1595, p-val = 0.0009"

#These next two lines show the Q-statistic with degrees of freedom as well as the p-value of the test. In this analysis, the p-value = 0.0009, suggesting that the included studies do not share a common effect size.

# Model Results:

#

# estimate se zval pval ci.lb ci.ub

# 0.1499 0.0316 4.7501 <.0001 0.0881 0.2118 ***

# Finally, we have the model results. The "estimate" provides the estimated model coefficient (i.e., the summary effect size) with standard error("se"). The z-value is the corresponding test statistic, "pval" is the corresponding p-value, "ci.lb" the the lower bound of the confidence interval and "ci.ub" the upper bound of the confidence interval.

#pred ci.lb ci.ub cr.lb cr.ub

#0.149 0.088 0.209 -0.037 0.325

# These two lines display the transformion of Fisher's z back to Pearson's r ("pred"), and the 95% confidence interval for r ("ci.lb" and "ci.ub") for reporting the meta-analysis.

#estimate ci.lb ci.ub

#tau^2 0.0081 0.0017 0.0377

#tau 0.0901 0.0412 0.1943

#I^2(%) 61.7324 25.2799 88.2451

#H^2 2.6132 1.3383 8.5071

#These four lines display estimates and 95% confience intevals for heterogeneity measures as descibed above.

# While the Q-statistic and I^2 can provide evidence for heterogeneity, they do not provide information on which studies may be influencing to overall heterogeneity. If there is evidence of overall heterogeneity, construction of a Bajaut plot can illustrate studies that are contribute to overall heterogeneity and the overall result. Study IDs are used to identify studies

b_res <- rma(yi, vi, data=dat, slab=study_id) # New meta-analysis with study ID identifier

# The next command will plot a Baujat plot.

baujat(b_res)

# Studies that fall to the top right quadrant of the Baujat plot contribute most to both these factors. Looking at the Molloy et al., 2014 data set reveals that 3 studies that contribute to both of these factors. A closer look the characteristics of these studies may reveal moderating variables that may contribute to heterogeneity

# A set of diagnostics are also available to identify potential outliers and influential cases.

inf <- influence(res)

print(inf)

plot(inf)

# The plot visualizes the printed dataset. As there are no studies are marked with an asterisk in the printed dataset, none of the studies fulfilled the criteria as an influential study.

# Now we visualize the meta-analysis with a forest plot.

forest(res, xlim=c(-1.6,1.6), atransf=transf.ztor,

at=transf.rtoz(c(-.4,-.2,0,.2,.4,.6)), digits=c(2,1), cex=.8)

text(-1.6, 18, "Author(s), Year", pos=4, cex=.8)

text( 1.6, 18, "Correlation [95% CI]", pos=2, cex=.8)

#We've created a plot with all 16 studies. Importantly, the correlations and 95% CIs are reported for each study as well as the summary effect size (the polygon at the bottom). The edges of the polygon represent the 95% confidence limit. Note the different sizes of each square - the studies with larger squares contributed more to the summary effect size.

### funnel plot

funnel(res, xlab = "Correlation coefficient")

#Tests for bias

regtest(res)

ranktest(res)

# Neither Egger's regression test (p = 0.3071) or the Rank correlation test (p = 0.3918) was statistically significant so there's no evidence of publication bias according to these tests.

#Trim and fill

# To demonstrate the trim and fill procedure now import a data set removing 3 studies ("dat_bias") with small effect sizes and high standard error. This command will only work if the "dat_bias" file is located in your working directory. If you don't know what your working directory is have a look at the R documentation https://cran.r-project.org/manuals.html (or search online).

dat_bias <- read.csv("dat_bias.csv")

View(dat_bias)

res.b <- rma(yi, vi, data=dat_bias)

res.b

confint(res.b)

# The next commands will print a funnel plot, perform Egger's regression test and the rank correlation test for the biased dataset.

funnel(res.b, xlab = "Correlation coefficient")

regtest(res.b)

ranktest(res.b)

#The trim and fill method imputes “missing” studies to create a more symmetrical funnel plot.

res.tf <- trimfill(res.b)

res.tf

funnel(res.tf, xlab = "Correlation coefficient")

# Moderators (all use a meta-regression model)

# Moderating effect of age

res.modage <- rma(yi, vi, mods = ~ meanage, data=dat)

res.modage

# The data under the "Test of Moderators" line provides the information needed. As the p value was greater than 0.05, this provides evidence that age did not significantly moderate the observed correlation.

# Moderator for methodological quality

res.modq <- rma(yi, vi, mods = ~ quality, data=dat)

res.modq

# As the p value was greater than 0.05, this provides evidence that methodological quality did not significantly moderate the observed correlation.

# Now, to look at a categorical variable. Here we look at the moderating effect of whether variables were controlled for.

res.mes <- rma(yi, vi, mods = ~ factor(controls), data=dat)

res.mes

# As the p value was less than 0.05, this provides evidence that controlling for variables significantly moderates the observed correlation

# Accounting for multiple effect sizes from individual studies using robust variation estimation

# First we import a data set aggregating the first 3 studies from the original dataset. As mentioed above, this command will only work if the "dat_mes" file is located in your working directory.

dat_mes <- read.csv("dat_mes.csv")

View(dat_mes)

# Calculate the effect size and effect size variances

dat_mes <- escalc(measure="ZCOR", ri=r, ni=n, data=dat_mes)

# Fit the meta-regression model with correction for small sample size

mes_ss <- robu(formula = yi ~ 1, data = dat_mes, studynum = id, var.eff.size = vi, modelweights = "HIER", small = TRUE)

print(mes_ss)

# The output confirms that there were 16 effect sizes from 14 studies. Analysis indicates a statistically significant point estimate [0.15; 95% CI (0.08, 0.22), p = 0.001].
